# Successful Reversal of Late, Severe Thrombotic Bioprosthetic Mitral Valve Stenosis With Anticoagulation Therapy

**DOI:** 10.7759/cureus.54556

**Published:** 2024-02-20

**Authors:** Juliet Galtes, Victor Gomez, Alejandro Sarria Arbocco

**Affiliations:** 1 Medical School, Florida International University Herbert Wertheim College of Medicine, Miami, USA; 2 Medical School, Nova Southeastern University Dr. Kiran C. Patel College of Osteopathic Medicine, Fort Lauderdale, USA; 3 Cardiology, Cardiovascular Wellness Center, Homestead, USA

**Keywords:** anticoagulation, apixaban, transesophageal echocardiography, bioprosthetic valve stenosis, bioprosthetic valve thrombosis

## Abstract

Late bioprosthetic valve thrombosis (bPVT) is a rare and potentially life-threatening complication following valve replacement with thrombus formation leading mostly to valvular stenosis or embolic phenomena. Clinically, it manifests as symptoms of fatigue, dyspnea, or reduced exercise capacity. The existing treatment guidelines lack clear recommendations for managing this specific presentation. In this case study, we present a distinct clinical scenario wherein the use of anticoagulation, specifically apixaban, successfully reversed very late, severe thrombotic stenosis in a 78-year-old woman with a Medtronic Hancock II porcine mitral valve (Dublin, Ireland). This case highlights the need to consider bPVT as an alternative etiology to valve degeneration in the setting of late bioprosthetic valve stenosis. Additionally, it demonstrates how apixaban therapy may serve as a viable treatment modality in these scenarios.

## Introduction

A rare complication of bioprosthetic heart valves is thrombosis, particularly when presenting late after implantation. This constitutes a serious event that can result in significant morbidity and mortality. Patients commonly present with subtle, increasing symptoms of fatigue and dyspnea, which, when left untreated, may evolve into overt left-sided heart failure [1].

A post-implantation echocardiogram, often referred to as a "fingerprinting" echocardiogram, is recommended within six weeks to three months following implantation [2]. This is crucial for establishing a post-operative baseline. In the setting of bioprosthetic mitral valves, various variables such as pressure half-time (PHT), transvalvular pressure gradient (PG), time-velocity integral (TVI) ratios, and effective orifice area (EOA) can be acquired and serially monitored to assess valvular dysfunction [2,3].

Scarcity of randomized controlled trials on the subject results in few, differing literature recommendations. Anticoagulants such as apixaban have proved effective in certain cases, including ours. This case report aims to encourage the consideration of apixaban as a treatment modality for bioprosthetic valve thrombosis (bPVT) in lieu of thrombolysis.

## Case presentation

**Figure 1:**
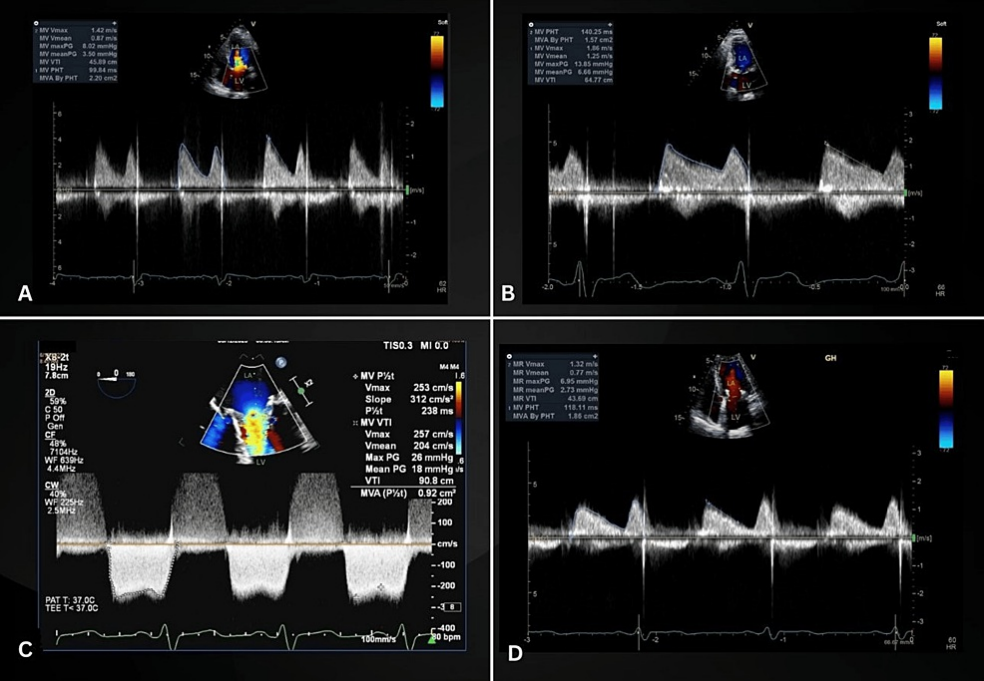
TTE and TEE detailing bioprosthetic valve hemodynamics (A). Baseline TTE obtained in 2018 demonstrating normal bioprosthetic valve hemodynamic. (B) TTE from January 2023 with a prolonged PHT and elevated mean transvalvular gradient. (C) TEE from June 2023 revealing further prolongation of the PHT and a marked elevation in mean PG. (D) TTE from July 2023, post-anticoagulation therapy with apixaban, revealing a complete reversal of the mentioned hemodynamic parameters to normal values TTE: transthoracic echocardiogram; TEE: transesophageal echocardiogram; PHT: pressure half-time; PG: pressure gradient; LA: left atrium; LV: left ventricle

A 78-year-old female with a past medical history of trigeminal neuralgia, type 2 diabetes mellitus, hypertension, and osteoarthritis of the right knee presents to our outpatient cardiology clinic in January of 2023 with fatigue and dyspnea. The patient had undergone a minimally invasive mitral valve replacement utilizing a 29 mm Hancock II (Medtronic, Dublin, Ireland) porcine mitral valve in January 2014 for the treatment of severe rheumatic mitral regurgitation. Medical records from an annual visit in 2018 revealed vital signs within normal limits and the absence of concerning physical exam findings. The latest biochemical data is from June 2022. Labs show hemoglobin, hematocrit, white blood cell count, electrolytes, liver enzymes, and lipid panel were all within normal range. Hemoglobin A1C, glucose, blood urea nitrogen, and creatinine were all high at 6.1, 116, 18, and 1.42, respectively. Figure 1A depicts a normal-functioning bioprosthetic mitral valve on a transthoracic echocardiogram (TTE). The expected valve function was supported by a mean transvalvular PG of 3.5 mmHg and a PHT of 99.84 ms in the absence of prosthetic valve regurgitation. Normal bioprosthetic valve leaflet excursion was documented at this time, as seen in Video 1.

**Video 1:** Baseline TTE Baseline TTE showing normal bioprosthetic valve leaflet excursion TTE: transthoracic echocardiogram

To evaluate the patient's new-onset symptoms, a repeat TTE was performed in January 2023. Figure 1B revealed a significant increase in the mean PG to 6.66 mmHg and marked prolongation of the PHT to 140.25 ms, indicative of prosthetic valve stenosis. Despite this, the mean PG did not reach the currently accepted cut-off of 9 mmHg to define a bioprosthetic mitral valve as severely stenotic in the presence of normal flow [2]. Although no significant mitral regurgitation was documented, leaflet motion appeared somehow restricted in comparison to her 2018 baseline study, as can be seen in Video 2. At that point, the working diagnosis was a degenerative disease of the bioprosthetic valve, and given the patient's functional class, it was decided to observe her. The patient was instructed to return if symptoms worsened.

**Video 2:** January 2023 TTE TTE showing restricted leaflet motion TTE: transthoracic echocardiogram

**Figure 2:**
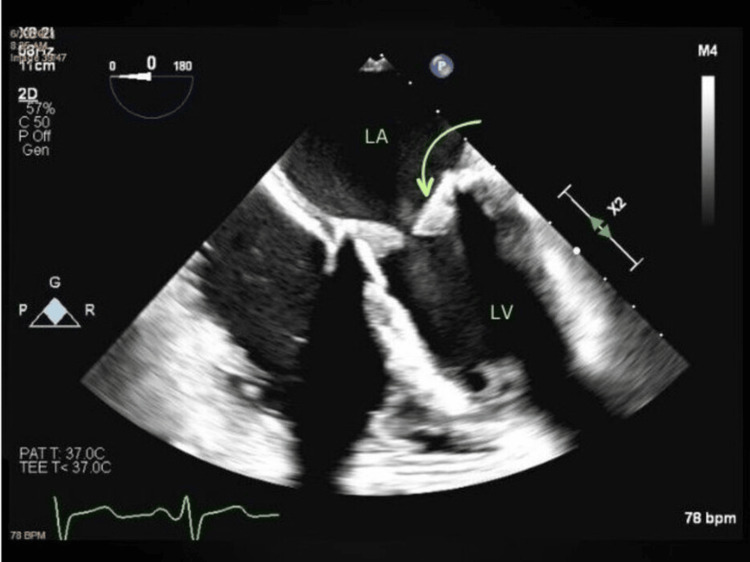
June 2023 TEE TEE from June 2023 showing a grossly thickened bioprosthetic mitral valve with echogenic material in the ventricular side of the leaflets (in green arrow) TEE: transesophageal echocardiogram; LA: left atrium; LV: left ventricle

In June of 2023, the patient returned with worsening fatigue, dyspnea, and markedly reduced exercise capacity. A transesophageal echocardiogram (TEE) was recommended to better assess her mitral valve prosthesis. TEE, seen in Figure 2 and Video 3, revealed turbulent flow across the valve as well as grossly thickened, restricted leaflets with clear echogenic material in the ventricular aspect likely secondary to in situ thrombosis. Figure 1C shows a drastically increased mean PG of 18 mmHg and a PHT of 238 ms, further supporting severe bioprosthetic mitral stenosis. A 3D TEE, seen in Video 4, allowed the appreciation of a markedly stenotic bioprosthetic mitral valve.

**Video 3:** June 2023 TEE TEE revealing turbulent flow across the valve as well as grossly thickened, restricted leaflets with clear echogenic material in the ventricular aspect likely secondary to in situ thrombosis TEE: transesophageal echocardiogram

**Video 4:** 3D TEE 3D TEE from June 2023 showing a markedly stenotic bioprosthetic mitral valve TEE: transesophageal echocardiogram

All of the above confirmed the diagnosis of late thrombotic bioprosthetic mitral valve stenosis. Cardiothoracic surgical consultation was requested while we opted to treat the patient with apixaban 5 mg, per oral, twice a day, for one month. We elected to use this dose as it is a standard therapeutic dose for this medication. Apixaban has a minimal effect on prothrombin time and partial prothrombin time; therefore, we did not measure these before initiating medication [4]. According to the 2020 American College of Cardiology/American Heart Association (ACC/AHA) Guideline for the Management of Patients with Valvular Heart Disease, for patients with a bioprosthetic mitral valve replacement, aspirin 75-100 mg and anticoagulation with vitamin K antagonist (VKA) are reasonable for three to six months post-operatively [5]. Long-term anticoagulation is not necessary unless the patients have another indication or comorbidity, such as atrial fibrillation. Our patient presented years after valve placement, and there are no clear guidelines for anticoagulation in late bPVT. We chose to use apixaban versus a VKA as there is no currently established guidance in cases like this one and the anticoagulant effect of apixaban is much more reliable and stable than a VKA.

At next month's follow-up, the patient conveyed a notable improvement in symptoms which prompted a repeat in-office TTE. Repeat imaging, seen in Figure 1D, illustrated the reversal of the bioprosthetic mitral valve physiology, supported by a decreased mean PG and PHT to 2.73 mmHg and 118.11 ms, respectively. Successful reversal of bioprosthetic valve stenosis with apixaban spared the patient from thrombolysis surgery. Following this, the patient was maintained on the current anticoagulation regimen indefinitely. Table 1 summarizes the patient's Doppler parameters of bioprosthetic mitral valve function at all time points discussed above. 

**Table 1:** Doppler parameters of bioprosthetic mitral valve function at different time points PG: pressure gradient; PHT: pressure half-time; TTE: transthoracic echocardiogram; TEE: transesophageal echocardiogram; ms: milliseconds; mmHg: millimetres of mercury

	TTE 2018 (baseline "fingerprinting")	TTE 01/2023 (at symptomatic presentation)	TEE 06/2023	TTE 07/2023 (post anticoagulation)
Mean PG	3.5 mmHg	6.66 mmHg	18 mmHg	2.73 mmHg
Mean PHT	99.84 ms	140.25 ms	238 ms	118.11 ms

## Discussion

The primary cause of dysfunction in bioprosthetic valves is typically attributed to structural deterioration, not thrombosis. Bioprosthetic valves are associated with a comparatively lower risk of thrombosis in comparison to mechanical valves, with reported incidences as low as 0.03% per year. This minimal risk has led to the consensus that long-term anticoagulation treatment is not typically warranted for individuals with bioprosthetic valves [6]. While bPVT has traditionally been perceived as a rare occurrence, recent data indicate a shift in this perspective, portraying bPVT as an increasingly acknowledged phenomenon [7]. The thrombotic risk is higher with the mitral valve compared with the aortic valve, and most cases are asymptomatic. A study by Egbe et al. described the overall thrombosis risk to be around 0.7-1.5%, of which the majority (65%) were identified >12 months after implantation, while 15% were identified five years after implantation. The peak incidence is reported to be around 13-24 months [8]. Our case represents an extreme of this spectrum, presenting nine years after implantation. The underlying principles of bPVT relate to disturbances in blood flow and activation of hemostatic factors. Artificial surface contact with blood and flow conditions around valve prostheses are associated with endothelial damage, vascular remodeling, and thrombosis. Furthermore, patient-related comorbidities such as renal insufficiency, obesity, diabetes mellitus, smoking, anemia, and low cardiac output states may predispose patients to thrombosis by promoting a hypercoagulable state [9].

TTE can define abnormal valvular hemodynamics, but TEE is superior in clarifying the precise etiology responsible for valve dysfunction [10]. An algorithmic approach has been suggested when evaluating mitral valve prosthesis with echocardiography. Current hemodynamic criteria suggest a PHT of >130 ms and a mean PG of >9 mmHg in order to define a bioprosthetic mitral valve as severely stenotic in the presence of normal flow [2]. Our patient clearly met these parameters, particularly at the time of TEE. 

Although the current 2017 ACC/AHA and European Society of Cardiology (ESC) guidelines recommend oral anticoagulation for only the first three months following surgical bioprosthetic valve replacement in the absence of risk factors, our case highlights the fact that the risk of bPVT is not limited to the first three months after implantation and should be suspected in the appropriate clinical scenario [11]. An agent, such as apixaban, may constitute an effective treatment modality in these cases. Apixaban is a novel oral anticoagulant that directly inhibits factor Xa in its free and bound forms. The antithrombotic impact of apixaban is achieved through the direct and selective inhibition of both free and clot-bound factor Xa. This inhibition of factor Xa results in a reduction of the conversion of factors II (prothrombin) to IIa, leading to a decrease in thrombin generation. It can be used in patients with non-valvular atrial fibrillation to reduce the risk of stroke and treat deep venous thrombosis (DVT) and pulmonary embolism (PE) [4]. Apixaban has various pharmacokinetic advantages including the absence of active metabolites, a predictable dose response, limited renal excretion, few interactions, and little need for therapeutic monitoring, making it an attractive treatment choice in patients with bPVTs.

Our case study illustrates the need to consider bioprosthetic valve thrombosis as an alternative etiology to valve degeneration in the setting of late bioprosthetic valve stenosis. Furthermore, it demonstrates how apixaban therapy may serve as a viable treatment modality in the proper clinical scenario. Limitations include the fact that this case may not be generalizable to the general population. There is limited data on the use of novel oral anticoagulants for the treatment of late-onset mitral bPVT, and it is difficult to establish a cause-effect relationship with strong evidence.

## Conclusions

In summary, this case contributes to the evolving landscape of bioprosthetic valve complications, urging clinicians to consider bPVT as a potential cause of late valve dysfunction and highlighting apixaban as a viable therapeutic option in the appropriate clinical context. Apixaban, with its favorable pharmacokinetic profile and established efficacy in other thrombotic conditions, emerges as a promising option for managing bPVT, challenging the notion that long-term anticoagulation may not be warranted in individuals with bioprosthetic valves. As our understanding of this phenomenon continues to evolve, further research and clinical experience will be essential in refining guidelines and optimizing the management of late bPVT.
